# One Health, many approaches: integrated vector management strategies support One Health goals

**DOI:** 10.3389/finsc.2025.1549348

**Published:** 2025-06-03

**Authors:** Hannah S. Tiffin, Jennifer R. Gordon, Karen C. Poh

**Affiliations:** ^1^ Department of Entomology, University of Kentucky, Lexington, KY, United States; ^2^ Bug Lessons Consulting LLC, Wheat Ridge, CO, United States; ^3^ Animal Disease Research Unit, Agricultural Research Service, United States Department of Agriculture, Pullman, WA, United States

**Keywords:** One Health, integrated vector management, vector control, pest management, vector-borne disease

## Abstract

Integrated pest management (IPM) laid the foundation for integrated vector management (IVM) by introducing strategies that prioritize prevention over intervention and the use of diverse management and control tools for arthropod vectors. Both approaches focus on reducing chemical reliance for management of targeted organisms by employing data-driven decisions and incorporating the use of additional non-chemical based management tactics. While IPM and IVM differ in their specific applications and management contexts, many of their fundamental principles remain the same. By diversifying prevention and control options, these management practices support healthier environments, animals, and populace, the three spheres of the One Health paradigm. One Health principles support and highlight the connections between animal, human, and environmental health and how these spheres influence one another. However, the connections and overlapping goals between IVM and One Health are infrequently discussed in tandem. This review will examine the numerous and varied methods of IVM for reducing global disease burden, detail the benefits of using IVM to achieve One Health goals, as well as benefits and considerations to incorporating a One Health lens with IVM.

## 1 Introduction

While pests and disease have been persistent challenges, cases of emerging and re-emerging diseases in humans, animals, and crops have increased in severity and frequency over the past half-century (1–3). The 20th century ushered in a new era of chemical, pharmaceutical, and technological innovations that reduced pests and increased agricultural output (4), as well as substantially improved public health outcomes with broad-spectrum antimicrobials and antibiotic interventions, insecticides, and sanitation reforms (5, 6). However, the belief that disease could be vanquished was soon recognized as a false prophecy. Nearly as quickly as chemical interventions were invented, resistance was noted (7–9), not only with modern insecticides and pesticides, but with other modern therapeutics such as broad-spectrum antibiotics and antiparasitics as well. Shortly after antibiotics became a cornerstone of modern industrialized agriculture, used both to prevent infections and promote growth rates in livestock, pharmaceutical resistance began to emerge. This resistance was observed in both the treated animals and farmworkers, with a strong correlation between antibiotic-resistant microbes in animals and the subsequent rise in resistance among humans (10, 11). The ongoing rise in pest and pathogen resistance, along with studies supporting the connection between human, animal, and environmental health, paved the way for the One Health concept to gain traction.

### 1.1 One Health origins

While the term “One Health” was officially coined in the 2000s, the concept itself dates back centuries. Initially termed “One Medicine” by Calvin Schwabe in the late 1800s, this concept was primarily focused on the connections between animal and human health (12). Historical examples, such as the early association of malaria with swampy environments (13), hinted at the critical link between environmental and human health long before mosquitoes were recognized as vectors of disease. In the late 19th to early 20th centuries, the pioneering work of scientists like Sir Ronald Ross and Major Walter Reed established that mosquitoes could transmit disease-causing pathogens to both humans and animals (12, 14), while Theobald Smith and F. L. Kilborne’s research on tick-borne cattle fever laid the foundation for understanding vector-borne zoonotic diseases (15). These studies were fundamental to our current understanding of pathogen transmission, aiding disease mitigation efforts by identifying that live organisms such as mosquitoes and ticks, or vectors, could transmit pathogens. Thus, vector management was developed, with the idea that by controlling the vector, one could control the pathogen and consequently, control disease.

With increasing recognition of the interconnectedness of animal, human, and environmental health, the One Health concept evolved. In the 1990s, the concept gained renewed attention in response to concurrent crises of increasing antibiotic and insecticide resistance and under-investments in public health infrastructure (11, 16, 17). With increased understanding of the importance of healthy ecosystems for healthy populaces, this concept was expanded to include three interconnected spheres (animals, humans, and the environment), and in the early 2000s the term “One Health” gained traction (17). This concept has become an integral framework for a multitude of agencies with extremely varied objectives regionally, nationally, and internationally to promote integrated approaches for addressing animal and zoonotic diseases and public health challenges.

### 1.2 Modern challenges in vector-borne disease management

Today, around 75% of emerging infectious diseases are zoonotic, with approximately 40% of known zoonotic viruses being vector-borne (18–20). Anthropogenic factors such as urbanization, land-use changes, industrial farming, and climate change have contributed to the increase and spread of vector-borne diseases by altering environments to favor vectors, like mosquitoes and ticks, and by increasing contact between humans and previously wild areas leading to disease emergence and spillover events (3, 21).

Anthropogenic manipulation of the environment has resulted in favorable changes for pest and vector populations to thrive. Increased emissions and accumulations of greenhouse gasses have contributed to environmental changes and an increase in global temperatures (22). These anthropogenic changes have made environments more suitable for pests by extending active seasons and expanding habitat ranges, thus increasing risk of vector-borne pathogen transmission in these areas (23–26). For instance, the number of suitable days in a year for mosquito activity has increased for many cities in the U.S. (27). Additionally, numerous areas in the U.S. have documented new invasive vectors (e.g., *Aedes japonicus* and *Haemaphysalis longicornis*) (28, 29) or expanding ranges of vector species (e.g., *Ae. aegypti*, *Ae. albopictus, Amblyomma americanum*, and *Am. maculatum*) (30–32). Compounding these challenges, widespread insecticide use has driven the rise of insecticide resistance in many vector species, further hampering control efforts. For example, large-scale deployment of insecticide-treated bed nets in Mali coincided with an increase in insecticide-resistant hybrid mosquito populations (7, 8). As broad-scale chemical interventions become more common, selective pressures have led to the spread of resistance genes and behavioral adaptations that enable vectors to survive control measures and have even led to the local extinction or extirpation of previously susceptible populations (7, 8). Furthermore, the change in environmental conditions has also included more destructive and frequent weather events (33), which can lead to mosquito-driven public health emergencies requiring rapid response by vector control professionals (34).

The effects and threats from vector-borne diseases are diverse and wide-ranging across local, regional, and global scales. Globally, mosquitoes remain the world’s most “deadly animal” contributing to the morbidity of millions and mortality of hundreds of thousands of people, primarily in tropical and subtropical regions from malaria and dengue infections. For instance, in 2022 alone there were approximately 249 million cases of malaria reported across 85 countries, resulting in 608,000 reported deaths (35). Even though the U.S. eradicated malaria officially in 1951 (36), imported cases are documented annually, and Texas, Maryland, and Florida all reported locally acquired cases as a result of imported cases in 2023 (37). In the U.S., tick-borne disease cases are also on the rise and account for approximately 75% of documented vector-borne disease cases (36, 38, 39). In 2022, public health professionals reported over 60,000 cases of Lyme disease (40). Spotted fever rickettsioses increased from fewer than 500 cases in 2000 to over 5,000 cases reported in 2019 before the case definition changed in 2020 (41). Additionally, new and expanding ranges of vector-borne diseases are being documented, such as the newly emerging threat of Oropouche virus to South America, Cuba, Europe, and North America (42). Other vectors and their associated diseases also impose high health and economic burdens, such as phlebotomine sand flies and leishmaniasis (43), kissing bugs and Chagas disease (44), and human body lice and typhus (45). Cases of vector-borne disease also incur heavy burdens on healthcare systems. For example, in the U.S., an estimated $1.3 billion is spent annually for Lyme disease (46) and $778 million over 14 years for West Nile virus (WNV) (47). Chikungunya virus can result in healthcare costs of approximately $14.8-33.4 million in the U.S. or $2.8 billion globally (48, 49).

Vector-borne diseases commonly affect animals as well. For instance, the prevalence of canine heartworm disease, caused by *Dirofilaria immitis* nematodes and transmitted by numerous mosquito species to domestic dogs and wild canines, has increased across much of the U.S. (50). Many tick species can transmit pathogens of significant medical and veterinary consequence, resulting in diseases such as Lyme disease (51) and Rocky Mountain Spotted Fever (52), which have the potential for zoonotic transmission to people in close contact with affected animals or infected ticks. Vector-borne diseases can cause devastating health and economic impacts to livestock as well. For example, approximately 80% of global cattle production is at risk of contracting tick-borne diseases (53). Ruminants such as cattle, sheep, and goats are at risk of infection from a high variety of bacterial, protozoan, and viral pathogens transmitted by ticks, causing economically important diseases such as anaplasmosis, babesiosis, and theileriosis (54). After eradication of bovine babesiosis in the U.S., the livestock industry has potentially saved $3 billion USD after accounting for inflation (55, 56). At a global scale, economic losses due to tick-borne bovine babesiosis and anaplasmosis can vary between $15–57 million USD, depending on the geographic location (57). While swine can suffer health consequences from vector-borne diseases such as spontaneous abortions and stillbirths, they also exemplify the disease risks from large-scale animal production practices and related invasive species (i.e., feral hogs) that can contribute to zoonotic outbreaks. One such example is the potential for a deadly Japanese encephalitis outbreak that could affect swine populations as well as horses, cattle, and people (58). Venezuelan, eastern, and western equine encephalitis are arboviral diseases that can kill unvaccinated horses after contracting the virus from a bite of an infected mosquito (59). Bites from arthropod vectors can also cause significant health effects, such as bite wounds or sensitivity reactions to salivary proteins, leading to well-known problems like “sweet itch” in horses typically associated with biting midges (60). Certain tick species can release salivary neurotoxins during feeding that can lead to tick paralysis, also known as tick toxicosis (61).

Vector-borne diseases affecting wild animals serve as another example of both disease consequence and disease transmission risk. For instance, in the southwestern U.S., seasonal outbreaks of plague occur in prairie dogs (*Cynomys* spp.) after contact with fleas infected with *Yersinia pestis*, with the potential for spillover into other species including humans (62). Globally, biting midges can transmit high-consequence pathogens in both domestic animals (e.g., African horse sickness virus in equines) and wild or farmed cervids [e.g., bluetongue virus in white-tailed deer (*Odocoileus virginianus*)] (63–65). Arthropods can further act as mechanical vectors by physically transferring pathogens from contaminated surfaces (e.g., manure, droppings, and other fecal material) to susceptible hosts. For example, a study conducted in an avian-influenza enzootic region in Japan found that blowflies (*Calliphora* spp.) may mechanically transmit high pathogenicity avian influenza (HPAI), a pathogen typically associated with direct contact or airborne transmission, between wild birds and farmed poultry, a finding with global significance given wild bird migratory routes (66). Additionally, recent detections of HPAI in U.S. dairy cattle and people in 2024 (67), underscore the interconnectedness of environmental, human, and animal health and the critical need for coordinated disease management strategies.

**Figure 1:**
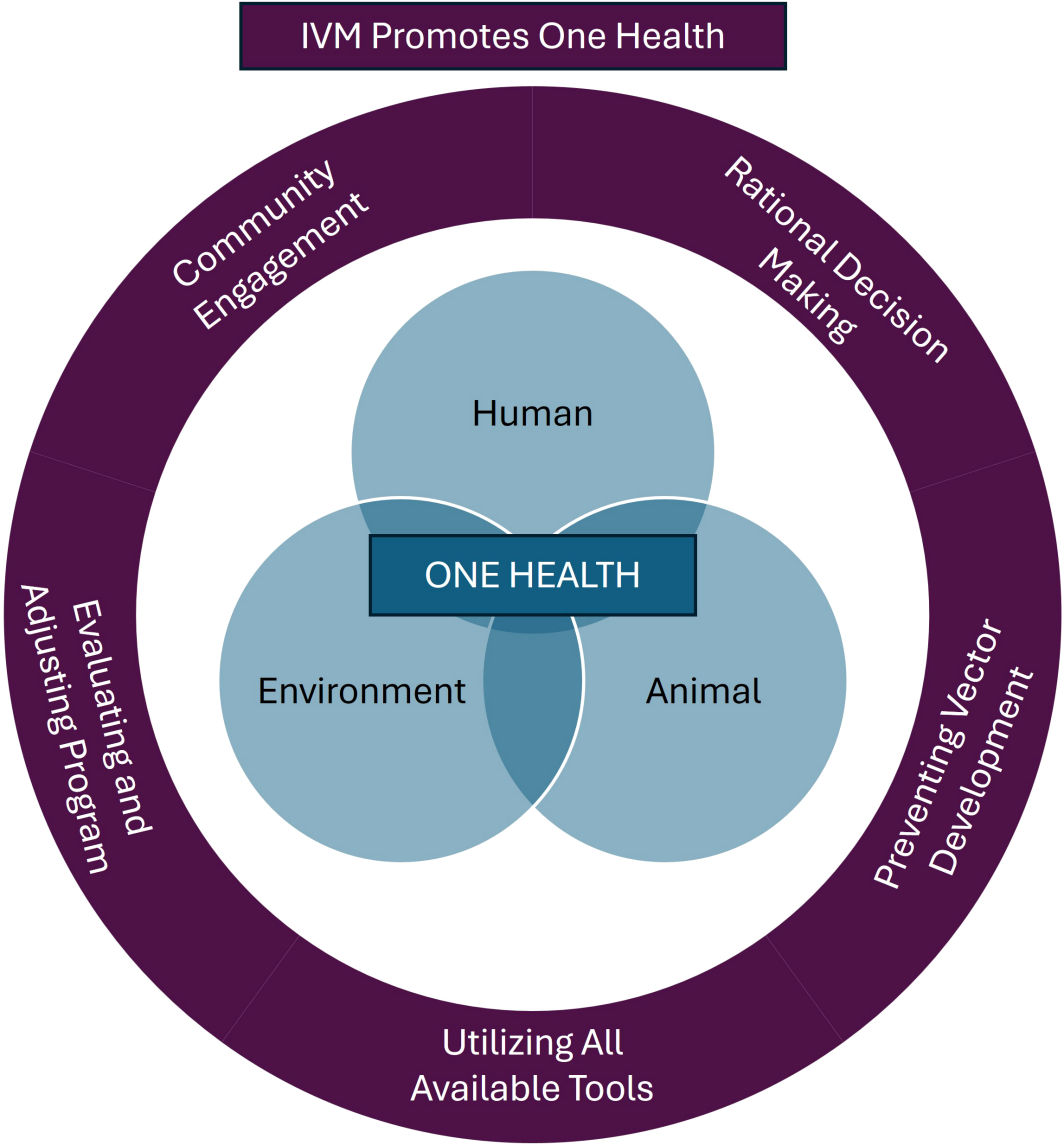
The integration of One Health principles within an Integrated Vector Management (IVM) plan. Integrated vector management promotes the use of all available tools to manage vectors and vector-borne disease with the ultimate goal of protecting human, environmental, and animal health when implementing and evaluating IVM plans. When designing an IVM plan, the three interconnected spheres of One Health should be considered in every step of the plan to ensure healthy people, animals, and environments.

**Figure 2:**
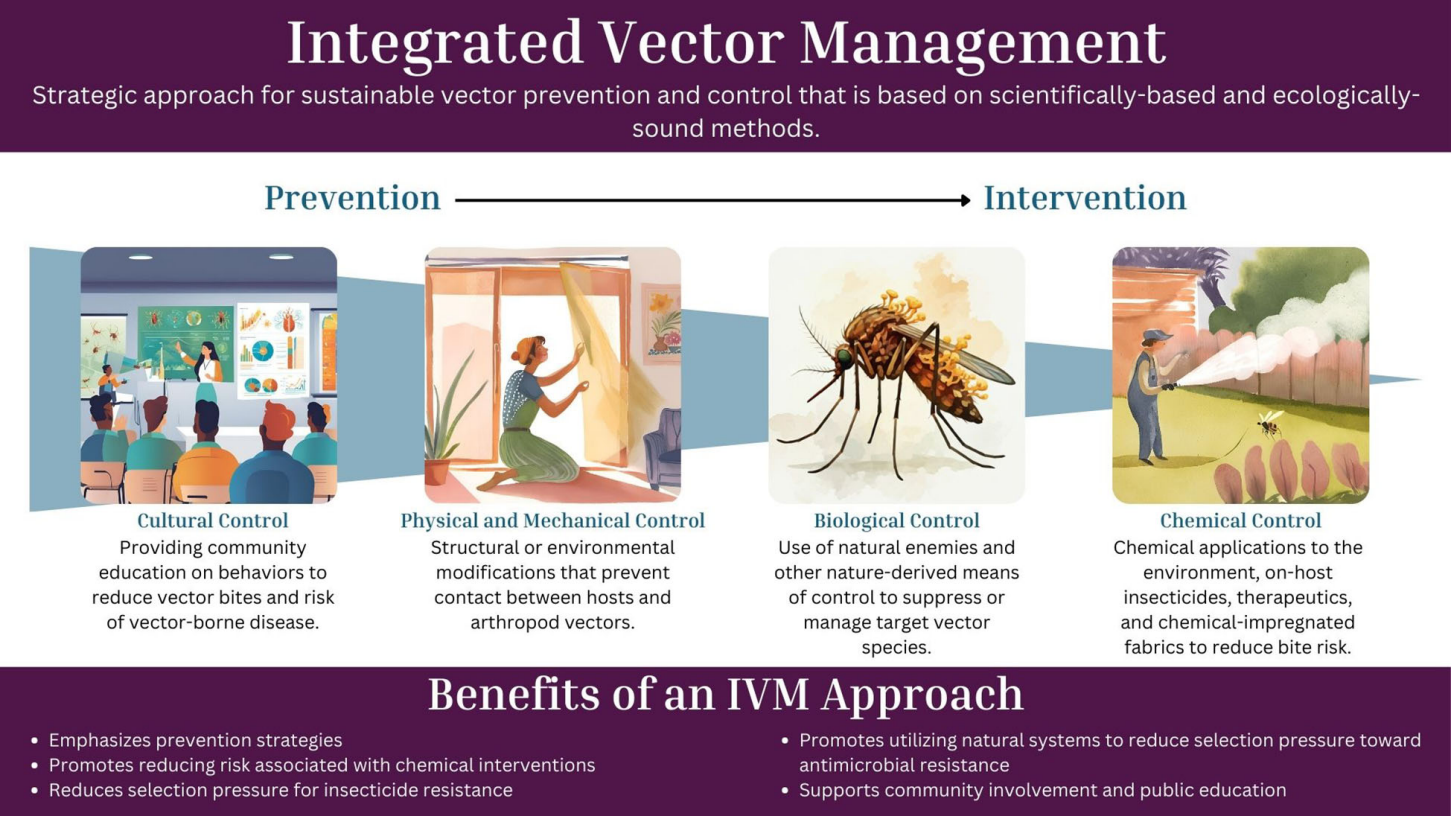
Management strategies involved in an integrated vector management (IVM) plan. Integrated vector management involves the use of multiple approaches for vector and vector-borne disease management. Moving from left to right shifts the focus from prevention-based tactics to intervention-based tactics, with the latter initiating control based on data and action thresholds. As a whole, IVM plans should incorporate diverse strategies to ensure a balanced and ecologically sound control approach. These management strategies fall under the “Utilizing all available tools” step in an IVM plan (**Figure 1**) and should be frequently evaluated and adjusted to changing needs.

To effectively manage vector populations and lower the risk of pathogen transmission, professionals can apply the One Health framework using science-based methods as part of an integrated vector management (IVM) program, a strategy adapted from integrated pest management (IPM) (**Figure 1**) (68). Integrated vector management promotes the use of all tools in a toolkit and emphasizes comprehensive planning and control strategies that go beyond chemical interventions and include cultural control, mechanical and physical control, biological control, and targeted chemical control when necessary (**Figure 2**). Community engagement is a critical component of an effective IVM program, ensuring that public health and pest control professionals work in tandem with local communities to detect and respond to vector-borne disease threats (69–71). Since professionals may not be able to survey or treat every location, educating the community on pest and vector identification, reporting, and disease risk reduction measures is essential to ensure the effectiveness of disease control programs. Additionally, IVM emphasizes data driven decision-making to guide control actions based on predefined action thresholds, mitigating non-target impacts, and evaluating efficacy to ensure vector control professionals achieve their goals of minimizing negative environmental effects (e.g., monitoring and managing insecticide resistance) (68). At the heart of a One Health-based IVM program, surveillance provides the foundation for vector control programs, providing baseline data to determine initial action as well as the data to evaluate changes to vector control programs (72–76). By incorporating the One Health framework into IVM, the health of humans, animals, and the environment can be safeguarded, ultimately reducing the risk of vector-borne diseases while minimizing harm to ecosystems.

## 2 From IPM to IVM: adaptations for vector-borne disease control

Within animal systems, vector-borne diseases have far-reaching effects, including direct economic losses due to mortality or decreased production in agricultural animals, direct and indirect effects within conservation and wildlife management contexts, and downstream disruptions in ecosystem services. The drivers and effects of disease are complex, involving interconnected systems that are not yet well understood, with the management and consequences thereof even less clear.

Based on the management strategies used in IPM, IVM utilizes similar tools within a vector and vector-borne disease management context (77). One of the major differences between IVM and IPM is that IVM decisions are not purely driven by economic thresholds. Instead, these decisions are also based on how resources can best be allocated to reduce the numbers of vectors or human cases, with the goal of preventing pathogen transmission (77). While different in motivations, IVM incorporates the same four core management strategies used in IPM (**Figure 2**):

1. Cultural control.
2. Physical and mechanical control.
3. Biological control.
4. Chemical control.

### 2.1 Integrated vector management control strategies

Cultural control refers to education, community engagement, personal protection behaviors, and sanitation efforts focused on the reduction of vector bites and risks of vector-borne disease. In public health contexts, this often involves community-level educational campaigns to raise awareness about local vectors and behaviors that can reduce disease risk, such as staying indoors during peak mosquito active hours or avoiding brushy and woody areas where ticks are more commonly found (78–80). In the context of animal disease, this can involve veterinarians educating pet owners about the benefits of vaccinations and prophylactics as well as risk-reduction behaviors to reduce the likelihood of bites (81, 82). In livestock systems, cultural control is often focused on sanitation practices that eliminate or reduce breeding conditions for pests and vectors, such as removal of manure for fly control or mowed pastures for tick control (83).

Physical and mechanical control can be broadly defined as structural or environmental modifications that prevent contact between hosts and arthropod vectors, thereby limiting pathogen transmission (84). While most studies extrapolate the use of barriers at the individual level, many of these barriers can be scaled up to protect entire communities or be geographically vast in scope, such as the use of draining trenches for mosquito control. By collectively implementing barriers, both individuals and those around them can be better protected. Examples of barriers include window and door screens, mosquito netting, landscape alterations, and environmental management that limit vector movement or survival. Additionally, wildlife fencing can prevent wild animals and any attached vectors from entering premises.

Biological control of vectors utilizes natural enemies (e.g., predators, parasites, competitors, and pathogens) to suppress and control the target vector species or host-derived advantages to reduce effects of vector bites or disease (e.g., anti-tick host resistance traits, harnessing and promoting effects of endemic stability). Specific examples of biological control to control arthropod vectors include the use of fungi, mosquitofish, parasitoids, arthropod predators, and vertebrate predators, with genetic or biological modifications of hosts or vectors themselves (e.g., using sterile insect technique and gene drives to limit vector or pathogen reproduction) often categorized as newer innovations within biological control. However, knowledge on the interactions between the agent, vector of interest, and the environment is needed to effectively use biological control agents as a sustainable control method and to mitigate potentially deleterious environmental consequences. This includes knowledge on the degree of host specificity, resources required for colony maintenance and release, and the possible ecological impacts following release of the biocontrol agents.

Chemical controls are used to prevent and reduce the risk of bites by eliminating the vector. This type of measure can include pesticide applications to the environment, on-host insecticides, acaricides, therapeutics, disinfectants, and chemical-impregnated fabrics. While there are some chemical applications used preventatively, chemical control is often used as an intervention in response to a specific event such as a positive index case (human or animal), positive vector (individual or pool), or vector abundance reaching a predefined action threshold (85–87). Some examples of chemical control include aerial applications of insecticides to control black fly or mosquito populations (i.e., aerial spraying or adulticiding), insecticide application to stagnant water sources (i.e., larviciding), and acaricide treated cotton in tick control tubes (i.e., host-targeted control).

Not all tactics fall exclusively within one strategy of IVM control, with numerous tactics harnessing the advantages of multiple control strategies. For instance, the use of insecticide resistant nets can be used as a physical barrier to prevent contact between hosts and mosquitoes, while also providing chemical control to kill mosquitoes that come in contact with the net (88). Another example is mowing pastures for tick control, which can be categorized as a cultural control and physical and mechanical control. This tactic generates a behavior to reduce viable habitat for ticks and it creates a barrier between high and low use areas, thus reducing tick bite risk (89). For more examples of control types in each management strategy of IVM, see **Table 1**.

**Table 1:** Integrated vector management (IVM) strategies, with examples of control within each strategy, the benefits and considerations for implementation, and recorded evidence of reductions in vector contact/bite in disease indices.

IVM management strategy	Control Type	Major vectors^1^ controlled	Benefits	Drawbacks	Evidence of reduction in bite or disease indices	References
Cultural control	Personal protection behaviors	Flies^2^, mosquitoes, ticks	Inexpensive; varied and flexible options (e.g., stay indoors during peak vector activity, wear long sleeves and pants outdoors)	Requires prior knowledge of local vectors; not always feasible (e.g., work outdoors during peak mosquito active hours)	Can reduce bites, but mixed results on disease reduction due to low uptake and behavioral adherence	78, 90–92
	Sanitation (e.g., manure management, remove organic material)	Flies, mosquitoes. ticks	Reduces vector breeding/immature development habitats (e.g., manure removal for flies, organic matter for mosquitoes, container emptying for mosquitoes); improves animal welfare and prevents pollution	Requires consistent implementation; may be influenced by farm infrastructure and labor constraints; may depend on socioeconomic status, especially for community-wide interventions	Associated with reductions in fly/mosquito breeding indices; lower larval indices for mosquitoes and reduced pest attraction; limited direct linkage to disease incidence	83, 93–98
	Community education campaigns	Mosquitoes, ticks	Promotes long-term behavior change; empowers local communities; children as change agents	Impact varies based on delivery method and population engagement; can require significant resources (labor, physical materials, etc.) to disseminate information	Linked to reduced vector habitats and increased adoption of personal protection behaviors	94, 99–102
Physical and mechanical control	Exclusion zones (e.g., window screens, mosquito nets, fencing)	Flies, mosquitoes, ticks	Varied options depending on vector system; low cost options available (e.g., netting over windows)	Potential for high costs depending on vector system (e.g., deer exclusion)	Reduces bites and disease transmission from fly and mosquito bites; mixed results on questing tick reduction from deer-exclusion; disease risk not assessed	103–108
	Water source reduction	Flies, mosquitoes	Targets immature stages; prevents breeding and/or immature development sites	Requires consistent community participation	Reduces entomological indices (larvae, pupae), but effectiveness varies depending on degree of community participation; disease outcomes less clear	93, 95, 96
	Vegetation management (e.g., mowed lawns, managed leaf litter, pasture management)	Mosquitoes, rodents (and indirectly any arthropods that they carry), ticks	May reduce questing tick habitat and limit host (e.g., small mammal) density in managed areas; reduces cryptic mosquito resting and immature development sites; may enhance visibility and safety; reduces tick habitat for survival; theoretically lowers contact between hosts (such as rodents) and arthropod vectors	Effectiveness varies by habitat, vector species, and maintenance frequency, which could mean several tactics are required to manage multiple species; could be labor intensive and expensive, depending on the amount of land that needs to be managed	May reduce vector-host encounters, especially with ticks and mosquitoes, but human disease outcomes are inconsistent; mixed or inconsistent impact on tick presence and density; no information on disease burden; more research needed overall	89, 109–112
Biological control	Vertebrate predators (e.g., mosquitofish/larvivorous native fish, opossum, guineafowl)	Mosquitoes, ticks	“Natural” control option; consumes some vector life stages	May not target all vector life stages; low specificity, where the predators’ diets do not solely rely on consuming arthropod vectors; risk of introducing invasive species that disrupt established natural ecosystems; potential for non-target effects; predators may also serve as hosts	No consistent reduction in mosquito or tick populations or disease risk; limited field evidence for efficacy; largely anecdotal or context-dependent	113–116
	Microbial larvicide (e.g., *Bti*)	Black flies, mosquitoes	Low toxicity to non-targets; effective for early control	Requires precise application and may need repeated treatments; limited to controlling immature stages in aquatic environments; cryptic habitats may be missed during application	Shown to reduce some vector-borne diseases such as like river blindness	117–119
	Entomopathogenic fungi (e.g., *Metarhizium anisopliae*, *Beauveria bassiana*)	Black flies, mosquitoes, ticks	Can infect a broad range of arthropods	May affect non-target arthropods due to broad host range; variability in fungal persistence and virulence depending on environment, production, formulation, and storage	Shown to reduce tick abundance (questing and on-host) and immature stages of flies; limited or no evidence for reduction in human disease incidence	120–126
	Host genetic manipulation or selective breeding	Biting flies, ectoparasites of livestock, ticks	May improve resistance to vector attachment or pathogen transmission; supports endemic stability	Expensive and time-intensive; limited to species with existing resistant traits or selection programs	Can reduce disease risk and reliance on antimicrobials in endemic areas	127–129
	Vector genetic manipulation (e.g., CRISPR, gene drives, RNAi)	Flies, mosquitoes, ticks	Targeted suppression or modification of vector populations; long-term potential	High initial cost (resources, labor, time, etc.); ecological unknowns; requires regulation and public acceptance; repeated releases often required; requires knowledge of vector genomes, which may not be readily available	Potential to reduce vector populations and transmission, though large-scale evidence is still emerging	128, 130
	Sterile insect technique (SIT)	Mosquitoes, screwworm, tsetse flies, other dipterans	Species-specific and environmentally safe; reduces reproductive success	Logistically demanding; repeated releases often required	Effective at population suppression, but disease impact varies	131, 132
	Parasitoid wasps and other arthropod predators	Flies, mosquitoes	Host specificity minimizes non-target effects; natural population control	Production and rearing are costly; survival and continual propagation of parasitoids depends on vector density and environmental factors	Can reduce vector abundance; limited data on direct human disease reduction	114, 133–135
	Reservoir host vaccination	Zoonotic pathogens in mammals (e.g., Lyme disease causative agents in rodents)	Breaks transmission cycle by preventing or reducing pathogen prevalence in hosts	Requires oral or bait-delivered vaccines; may be expensive at scale; baits may be uptaken by non-target animal hosts	Reduces pathogen prevalence in vectors; potential downstream reduction in human/animal cases	136–138
Chemical control	Host-targeted control (e.g., acaricide-treated cotton, on-animal products)	Fleas, flies, mites, ticks	Reduces vector interactions with hosts; interrupts transmission cycles	Uptake depends on availability, cost, and user compliance; could be costly, depending on the type of control	Shown to reduce questing and on-host tick abundance; reduced fly pressure on equines	123, 139–144
	Ultra-low volume (ULV) adulticiding	Mosquitoes	Precise application; targets active mosquitoes with minimal chemical use	Dependent on vector biology and environmental conditions; public concern over exposure; insecticide resistance possible	Can effectively reduce adult mosquitoes when timed correctly; indirect impact on disease	145, 146
	Targeted larviciding	Mosquitoes	Cost-effective especially in urban areas; prevents adult emergence	Limited to accessible aquatic sites; may require repeat applications; cryptic habitats may be missed during application	Reduces vector populations	147, 148

^1^In the context of medical and veterinary health, “vector” refers to biological and mechanical vectors as well as pests that cause health distress, such as non-vectorial flies.

^2^Even though both are dipterans, flies and mosquitoes are categorized separately to reflect differences in their ecology, medical and veterinary health relevance, and control strategies.

### 2.2 Historic use of IVM practices

Historically, humans have manipulated their environment to manage vectors and vector-borne disease. Long before the new era of chemical control in the 1900s, people were exploring different methods of vector control. One of the first intervention trials was conducted in the 1800s in Rome and used physical barriers to prevent pathogen transmission from vectors. The trials, conducted by Angelo Celli, found that covering windows and doors with cloth or screening to prevent mosquitoes from entering homes led to a reduction in malaria cases (149, 150). Because infection frequently occurred indoors, several studies and reviews successfully implemented simple modifications to homes, such as using netted windows, screened doors, and closed eaves, to protect people from Anopheline mosquitoes and reduce the number of clinical malaria cases (88, 150–153).

Agricultural systems were one of the primary innovations that enabled the rise of complex human societies, bringing crops, animals, and humans closer together in higher densities. These changes quickly led to changes in pathogen transmission cycles, often leading to increased rates of zoonotic pathogen transmission due to crowded and unsanitary conditions as well as increased human-animal contact and densities (154–156). In fact, many diseases with high global consequence likely arose directly or indirectly due to farming and domestication of animals, such as malaria, measles, smallpox, and influenza (3, 154–156). While not all of these diseases are vector-borne, many can be transmitted by arthropod vectors, allowing for transmission to occur outside of direct animal-to-human or human-to-human contact.

Synthesized chemicals have been used as a vector control tool since the early 1900s (76). As chemical use became more common and widespread, so did the consequences. As a result, public opinion on chemical use for vector control has been controversial, with many successes marred by deleterious downstream effects. For example, in the past, public health professionals widely and indiscriminately used the pesticide dichloro-diphenyl-trichloroethane (DDT) to control vector-borne diseases, such as malaria and typhus, with much success (157). Unfortunately, the egregious use of this pesticide also led to the rapid development of insecticide resistance and other negative consequences such as long environmental persistence, accumulation in fatty tissues, declines in wildlife health, and dispersal through the upper atmosphere (157).

Today, IVM programs and the decisions to implement vector and vector-borne disease control are data-driven. As such, IVM programs conduct routine surveillance of pest populations and pathogen activity to inform control decisions as well as to collect efficacy data to improve future iterations. Modern technological advancements also enable the use of precision equipment and techniques to apply an optimal amount of pesticide at targeted delivery sites for direct contact with the vector. For example, ultra-low volume (ULV) space sprays target adult mosquitoes and use very small volumes of pesticide (generally <1 oz/acre) (145). While delivery often involves precision tools, chemical interventions can still negatively affect non-target organisms such as terrestrial wildlife, aquatic species, and beneficial insects (146, 158–161). This highlights the significance and need for continual surveillance and evaluation of IVM programs in order to maximize the impact on entomological and disease indices while minimizing harm to the environment and its inhabitants.

## 3 IVM in a One Health context

Humans and animals live together in one environment, and the interactions among the three groups can influence the overall health experienced within each group (162–164). As such, One Health emphasizes the importance of protecting and maintaining a healthy environment to reduce the incidence of disease in humans and animals. An IVM program supports One Health’s goal of preserving health across people, animals, and the environment by routinely engaging communities, surveying the environment for vectors and pathogens to make data-driven control decisions, and continuously evaluating effectiveness of management practices to ensure goals are met and modify strategies as needed (**Figure 1**). Specifically, IVM focuses on the prevention of vector bites and the transmission of vector-borne pathogens, encouraging community involvement to reduce vector encounters, minimizing pressure that could lead to insecticide and antimicrobial resistance, and reducing reliance solely on chemical controls.

While vector control strategies, policies, and methodologies have significantly reduced the burden of vector-borne diseases in many regions, the field remains largely confined to a subset of human health professionals, such as vector and pest control operators, vector biologists, entomologists, public health scientists, and policy-makers. This discipline-specific focus, while often effective in addressing immediate public health concerns, limits the broader potential of IVM to enhance not only human health but also animal and environmental health and wellness. By expanding the scope of IVM through a One Health framework, interdisciplinary collaboration can be strengthened to include professionals in veterinary sciences, wildlife and environmental sciences, data sciences, social and behavioral sciences, conservation and preservation professionals, educators, mental health experts, and policy-makers involved in these varied sectors. This holistic and collaborative approach would enhance efforts to monitor and mitigate animal and human disease outbreaks (including zoonotic diseases and spillover cases), improve behavioral change and adoption of prevention tactics, reduce environmental pollution and mitigate environmental indirect effects of chemicals and pharmaceuticals used for disease prevention and control, and promote ecological resilience (165). Furthermore, fostering healthier ecosystems can yield indirect benefits to human health, such as improved mental well-being through increased access to natural spaces and reduced environmental stressors.

Beyond interdisciplinary cooperation, a One Health-based IVM strategy enables more effective surveillance, ecological interventions, and sustainable vector control. Integrated disease monitoring across human, animal, and environmental health sectors can provide early detection of emerging vector-borne disease threats, allowing for timely intervention. Ecological approaches, such as habitat restoration and biological control using natural predators, can reduce reliance on chemical interventions, slowing insecticide resistance and minimizing harm to non-target species. Additionally, coordination between health disciplines and professionals can enable agile future adaptation, planning, and response efforts. For instance, coordination between vector control professionals, climate scientists, and urban planners could facilitate developing climate-smart vector management to prepare for shifts in vector populations due to changing environments and other factors. By incorporating these broader considerations into vector management programs, IVM can continue to push public health beyond a reactionary model toward a proactive, adaptive, and ecologically responsible approach that supports all three sectors of health - human, animal, and environmental.

Additionally, by using a One Health approach to IVM, the health of one sector can safeguard the health of the other sectors. For example, biosecurity and surveillance measures in poultry houses targeting avian influenza not only reduce transmission among birds but also minimize risks to farm workers, other livestock, and potentially wildlife as well. Similarly, the re-emergence of bed bugs (Cimex lectularius) in poultry facilities underscores the need for coordinated management strategies, as infestations pose risks to both poultry workers and poultry production systems (166–168). Additionally, in zoonotic disease systems, reducing disease incidence in animal reservoirs and alternative animal hosts can lower human infection risk. For instance, treating domestic pigs and dogs for malaria parasites can decrease the malaria infection risk to humans under certain circumstances (169). Similarly, in the Lyme disease system of the northeastern United States, interventions targeting reservoir hosts have shown promise in reducing human risk. Methods such as treating mice with permethrin to eliminate ticks (139, 140, 143) or vaccinating wildlife hosts against *Borrelia burgdorferi* (the causative agent of Lyme disease) and other tick-borne pathogens (136, 137) can lower the prevalence of infected ticks, thereby decreasing the likelihood of pathogen transmission to humans (89). For many zoonotic and vector-borne disease systems, effective management and control requires education and intervention across multiple sectors.

### 3.1 IVM promotes prevention strategies

“A dose of prevention is worth a pound of cure,” is a core tenet of IVM by emphasizing prevention of vectors and vector-borne disease over intervention when feasible. Successful prevention of vectors and vector-borne disease often combines several preventative strategies such as community education, behavioral changes, and mechanical and physical barriers that include landscape and environmental modification. With rising concerns on the potential adverse health and environmental effects of chemicals used to control vector populations, prevention-focused tactics, such as cultural control and physical and mechanical barriers, can be used as an alternative or a supplement to chemical controls (79, 170, 171). Vector knowledge, risk reduction behaviors, and sanitation serve critical roles in any effective IVM plan regardless of industry (e.g., public health or agriculture), thus many IVM programs start with cultural control and habitat modification to prevent vector development. For instance, one of the most important prevention methods in livestock systems is to ensure that manure is handled quickly, efficiently, and safely, as manure attracts many arthropod species (97). Inappropriately handled manure not only contributes to pest problems and animal welfare concerns on-farm but can also lead to downstream water and land pollution, affecting environmental and human health as well (98).

Mosquitoes can also benefit from unkempt environments because their immature life stages require a water source and organic material for development. As such, one critical mosquito control strategy is source reduction, such as removing or emptying containers that hold water or even laser leveling the ground. Programs that include source reduction and covering containers in their educational program can directly impact entomological indices such as reductions in larvae and pupae (93, 95, 96, 172–174). However, these reductions are not always consistent due to a variety of factors ranging from community socioeconomic status to behaviors of cryptic mosquito species (94, 175–177).

Physical barriers can provide protection from vector bites thus reducing the risk of pathogen transmission without using chemical controls. For example, even non-treated bed nets prevented a substantial proportion of Anopheline mosquitoes from entering homes in a study comparing treated and non-treated bed nets (106). More significant modifications such as building raised homes, platforms, and seating areas above the ground have also been successful in preventing mosquito bites, since Anopheline mosquitoes tend to host-seek closer to the ground (103, p. 198; 105, 107, 108).

Like altering the home or facility itself, the environment and landscape surrounding the home, community, or livestock facilities can be modified to prevent the entry of arthropod vectors and hosts relevant to vector-borne disease cycles. For instance, public health professionals have suggested modifications such as installing physical barriers around a lawn or adding ground barriers to separate properties from forested regions to prevent ticks from entering areas frequented by people (112). Efficacy of these fences and ground barriers to deter ticks will differ depending on several factors such as the tick-specific behaviors, tick-host associations, host behavior and size, and physical properties of the barriers (89, 104, 109–111, 178–183).

General environmental and landscape management removes potential arthropod vector habitats in areas that may be used by people and animals, reducing contact with vectors. Theoretically, unmanaged yards that have trash, leaf litter, or unmowed grass can provide additional habitats for ticks and small mammal hosts such as white-footed mice (*Peromyscus leucopus*) (112). However, the consensus on whether regular lawn management has any effect on tick presence or abundance is unclear. While trash predicted higher abundance of questing larval *Ixodes scapularis* ticks in New York (109), leaf litter accumulation and unmowed properties did not result in significant differences of nymphal *I. scapularis* ticks on properties in Connecticut (111). Differences are also likely dependent on the biology and behaviors of the tick species and life stages of interest (184–191). Field evaluations on landscape management to reduce tick abundance are scarce and even fewer studies have evaluated these methods as ways to reduce tick-borne disease incidence (89).

### 3.2 IVM supports community involvement and public education

Implementing community-wide educational efforts with the goal of effecting long-term behavioral changes to prevent vector bites and pathogen transmission has been successful when integrated with other methods. For example, Fonseca et al. (192) attempted to reduce *Ae. albopictus* abundance by using an IVM program that targeted multiple mosquito life stages through a combination of active source reduction, adulticiding, larviciding, and public education. The study found that combined outreach education and community-wide source reduction resulted in significantly less *Ae. albopitcus* populations, noting that the combined efforts were more effective and lasted longer than adulticide spraying alone (192). Likewise, a similar study conducted in Mexico also reported that educational campaigns were more effective at reducing Ae. aegypti populations compared to spraying adulticides (193). In Africa, an IVM program that included long-lasting insecticide treated nets (LLINs), larviciding with *Bti*, and community engagement reduced the prevalence of malaria by 50% in an environment with low infection prevalence (80).

For IVM, educational campaigns can take many forms (e.g., outreach events, presentations, publications, school lessons, and workshops) and have been distributed by several types of groups including mosquito and vector control programs, academic institutions, extension programs, non-profit organizations, and private entities. The goal for many of these programs is to inform and mass-educate communities, whether they are emphasizing personal prevention measures, the risks of vector-borne diseases, or interventions and products designed to prevent vector-borne disease transmission. The general principle is that when more people are informed about the disease system and associated risks and are given options for prevention, they feel more empowered to make informed decisions about their preventative behaviors (78, 194). Although community acceptance of prevention practices may be high or perceived positively, successful adoption of these behaviors also requires intersectoral collaboration, government policy, and financial support (151).

In addition, educational programs should also include information on emerging tools and products that stakeholders can incorporate into their vector-borne disease prevention or management plans. Understanding community engagement practices and innovation characteristics that enable or hinder adoption of products can improve sustainability and acceptance of new products. In Guatemala, intersectoral collaborations with communities led to the development of a community-based IVM plan for Chagas disease prevention, leading to successful adoption and acceptance of their integrated strategies (195). Further, innovations that are lower in complexity and compatible with local practices are likely to have better acceptance amongst stakeholder groups (195, 196). “Farmer field schools” in Sri Lanka developed and presented curricula focused on emerging IVM tools and practices and emphasized the risks of using chemical control in agriculture (197). Overall, this led to successful adoption of several practices related to improving environmental sanitation and personal protective measures against mosquitoes. While a foundation of knowledge of vector-borne disease systems is important, education on the application of new technologies and aligning those technologies with local contexts, values, and needs of the participants is critical in ensuring successful adoption of new behaviors or breakthrough technologies.

The form of information delivery is just as important as the information itself, as engaging information is more likely to connect with and motivate people to take preventative measures and participate in efforts to reduce the risk of vector-borne disease. Passive education and information delivery is often ineffective. Instead, educators should aim to identify the best method of information dissemination for their audience and co-develop campaigns with the community and their leaders or peer educators (69, 94). One example of a method that has been effective at information dissemination is the integration of vector and vector-borne disease messaging in school curricula. Children attending school are considered change agents since they can communicate health messages to their families, peers, and the greater community, therefore, integrating vector and vector-borne disease educational programs into school curricula has been successful in providing far-reaching community-wide education (99, 198–201).

### 3.3 IVM promotes utilizing natural systems to reduce selection pressure toward antimicrobial resistance

Rising global population, urbanization, and increasing demand for animal products have driven a significant expansion in livestock production. This intensification, particularly in high-density animal operations, has both directly and indirectly exacerbated the challenges of emerging and reemerging pests and diseases, including their rapid transmission and difficulties with containment (202, 203). To curb or prevent disease transmission, including vector-borne diseases, animal husbandry professionals have dramatically increased their use of broad-spectrum antibiotics, antimicrobials, and anti-parasitics across entire herds and flocks, often without regard to the current or potential disease status of individuals or the population (204). Antimicrobials are also used at subtherapeutic doses for disease prevention and growth promotion purposes (204). In fact, animal production operations use the vast proportion of the global antimicrobial supply (3, 205, 206). The heavy use of antimicrobials in animal production has led to increasing rates of antimicrobial resistance in various pathogens that threaten not only livestock and animal health, but human health as well (3, 4, 11, 98, 203).

It should be noted that while antimicrobial resistance is still a major issue for many countries, antimicrobial usage in the European Union (EU) has been decreasing since 2022 when the new Regulation on Veterinary Medicines Products (Regulation EU 2019/6) was implemented in an effort to curb antimicrobial resistance in agricultural animals (207). For some countries such as Sweden, Finland, Denmark, and the Netherlands, bans on antimicrobials for animal growth purposes started prior to the 2022 regulation, and these countries have argued that restrictions on non-therapeutic uses of antimicrobials can be implemented with minimal production consequences (208, 209). Instead of relying on antimicrobials, these countries emphasize that the health of livestock should rely on effective husbandry and welfare practices (210). Given that the regulations only started in 2022 across the EU and that each country will have their own policies to manage the ban, further research and analyses are required to fully comprehend the long-term effects of antimicrobial bans across the EU (211, 212).

The concept of endemic stability can also be incorporated into an IVM plan as a possible disease management tool. Endemic stability describes a state where a pathogen is present within a population but rarely causes severe disease, often due to early exposure that confers lasting host immunity (4). This phenomenon is typically observed in regions with a long history of host-pathogen coevolution. For example, in East and Central Africa, bovine theileriosis is endemic, and cattle are consistently exposed to both the pathogen and tick vector species. In these regions, indigenous Zebu cattle (*Bos indicus*) frequently demonstrate immunity, with minimal or no clinical signs of disease, exhibiting endemic stability (127, 129). Additionally, studies of Zebu and other indigenous African cattle breeds’ host defense mechanisms may provide genetic and immunological traits that are advantageous and could be harnessed for use with cattle breeds that are less resistant and resilient to tick bites and tick-borne diseases (128, 213).

Historically, interactions between wildlife and livestock can lead to disease transmission or spillover between species. This can occur when wildlife serve as reservoirs for naturally-circulating pathogens, thus facilitating transmission of vector-borne diseases in areas of wildlife-livestock contact and overlap (214). However, wildlife diversity serves beneficial purposes as well, even for the livestock industry. Whereas ticks may feed on a variety of host species, some hosts may be better able to remove or kill ticks than others and thus may serve as “trap” species for ticks, thereby reducing overall parasite abundance in that particular community and reliance on antiparasitics or antimicrobials (215). However, studies exploring the relationship between biodiversity and disease transmission cycles have yielded mixed results, highlighting the complexity of these interactions (214, 215) (see section 4.1).

Methods that eradicate parasites, pests, and diseases often only work effectively in the short-term. For example, if the same or a similar pest (re)emerges, the host may no longer have immunity against infection or infestation and is at greater risk of morbidity and mortality (216). The increasing rates of antimicrobial resistant infections reveal the weaknesses in relying solely on therapeutic and chemical intervention as management strategies. While an IVM strategy may not resolve the current antimicrobial crisis, its value is highlighted by promoting diverse strategies and using surveillance to detect and predict disease threats before they occur, ideally reducing the reliance on antimicrobials for preventative and therapeutic interventions (4).

### 3.4 IVM promotes reducing risk associated with chemical interventions

Of 84 surveyed countries (not including the U.S.), the vector-borne diseases linked to the highest pesticide use by public health professionals were malaria, dengue, leishmaniases, and Chagas disease (217). These diseases alone account for 107–408 million infections and over 600,000 deaths every year worldwide (218–221), clearly highlighting the need for effective vector control. However, even though pesticides are one of the most valuable tools available for vector control and disease risk management, they also carry some of the greatest hazard (potential to harm) and risk (the likelihood harm will occur) to the health of people, animals, and the environment.

As such, an effective IVM plan would utilize insecticides to prevent and reduce vectors as only one tool in a larger toolkit and their use is based on data-driven decisions and action thresholds (68, 222, 223). One of the goals of using numerous control tactics and pre-planned, data-informed insecticide applications is to decrease use of insecticides overall, lower incidence of insecticide and behavioral resistance in target vector populations, mitigate non-target effects, and reduce environmental contamination from chemical use. For instance, in an agricultural setting, by using an IPM approach while growing corn and watermelon, farmers applied 95% fewer pesticide applications compared to using a standard conventional management approach and were still able to maintain and even increase crop yields (224). Unfortunately, there are few, if any, studies in vector systems that have done similar comparisons. Future research to assess changes in insecticide use and associated costs using IVM compared to other vector control methods would provide critical information for developing efficient and cost-effective vector management plans.

When using chemical control tools, many IVM programs choose lower risk strategies before progressing to relatively higher-risk options. For instance, science-based mosquito control programs will target immature mosquitoes as a first-line measure with the goal of preventing adult emergence. While costs vary substantially, larviciding is particularly cost-effective in urban areas where targeting fewer aquatic sites can protect more people from mosquito bites and associated diseases (147, 148). Several active ingredients exist to perform this action such as methoprene, pyriproxyfen, and spinosad (68). Another tool used by mosquito control professionals, *Bacillus thuringiensis israelensis*, or *Bti*, is a bacterium with spores that produce toxins lethal to immature mosquitoes, black flies, and fungus gnats (117). In addition to being used in mosquito control programs, *Bti* has also been successfully used as part of an IVM plan to reduce cases of human onchocerciasis, or river blindness, by controlling black flies that can transmit the causative agent, *Onchocerca volvulus* (118, 119, 225, 226). The specific action of *Bti* products, combined with their low toxicity to both mammals and honey bees, makes them excellent tools for managing vectors while minimizing non-target effects (117).

Control measures that eliminate vectors but also hold the potential to impact non-target organisms, such as pollinators, may be too risky for the reward of vector elimination. Thus, to further minimize the exposure of non-target organisms, vector control professionals must understand the biology of both the targeted pests and other organisms in the environment to help ensure that applications are timed to affect the vector while minimizing impact on non-target organisms. Many mosquitoes targeted by vector control professionals are most active at dusk and night (227–229) while other organisms, such as bees and butterflies, are more active during the day (131, 230). Taken as a whole, strategically timed ULV applications of adulticides intended to target mosquitoes are often applied at dusk and early evenings. As a result, the tiny droplets float in the air and come into direct contact with active, flying mosquitoes while ideally avoiding non-target organisms at rest, such as pollinators.

### 3.5 IVM reduces selection pressure for insecticide resistance

As previously described, pesticides can manage vectors in the environment, but sometimes their use can cause counterintuitive impacts such as selecting for insecticide resistant vectors. Application of pesticides to control vectors exerts selection pressure by killing individuals susceptible to the pesticide, while survivors become the progenitors of the next generation (217). If this pressure persists, the population may become resistant to the insecticide, leading to control failure. Researchers worldwide have documented insecticide resistance for many different vectors including mosquitoes (231–233), ticks (234–236), body lice (237, 238), kissing bugs (239), fleas (240, 241), and sand flies (43).

A proactive vector control program includes insecticide resistance monitoring of the targeted vector populations to mitigate risks and take appropriate actions to manage insecticide resistance when it occurs (242). When resources permit, programs should also investigate the underlying molecular mechanisms of resistance as this will inform what insecticide resistance management (IRM) strategy may be most effective at delaying or reversing resistance. Potential IRM strategies include rotating between different modes of actions, using multiple modes of action simultaneously, and targeting different life stages for control (68). These strategies can be effective because the mechanisms of resistance often carry a fitness cost, therefore, removing the selection pressure can lead to a reversion back toward susceptibility (243). By regularly monitoring and managing insecticide resistance, IVM reduces selection pressure for resistance (244) and subsequently helps manage the environmental impact of insecticides.

## 4 Considerations when implementing and evaluating IVM programs

As exemplified by many of the studies highlighted in this review, IVM programs and efforts have had great success in vector and disease reduction across many disease systems, communities, and regions. However, the success of an IVM program is often dependent on the unique needs, locality, resource availability, and involvement of the community and solutions available for the vector-borne disease system. These varied and diverse considerations make generalizations from study results challenging to interpret and implement in different contexts. This is particularly apparent with studies that investigate interventions or methods employed at the community level or that require field evaluations, highlighting the complexities in evaluating and implementing effective IVM strategies for disease and vector reduction.

### 4.1 Ecological principles cannot be applied broadly due to complexities in vector-borne disease systems

Disease systems are inherently complex, with vector-borne diseases particularly complex as they involve multiple live organisms in the pathogen transmission and maintenance cycles. With several organisms represented in these systems, the role of biodiversity in enzootic cycles of vector-borne disease is still not well-understood. Human manipulation of the environment can lead to decreased biodiversity (245), which can contribute to increased risk of vector-borne disease in certain circumstances. For instance, the dilution effect posits that lower species diversity can lead to higher incidence of vector-borne disease through increased likelihood that a vector will bite a reservoir host rather than a dead-end host without disease transmission potential (246).

An example of the dilution effect in action is the Lyme disease system. Ixodid ticks transmit *B. burgdorferi*, the causative agent of Lyme disease, which is the most common vector-borne disease reported in the U.S. (40). The primary vector, *I. scapularis*, is a three-host tick, meaning that each life stage requires a blood meal from a different host to molt into the next life stage (247). Given this tick species’ generalist feeding habits and the varying degrees of reservoir competency for different animal hosts, the dilution effect predicts that environments with higher biodiversity (i.e., more diverse host options) should present lower risk of Lyme disease pathogen transmission since tick bites on hosts with lower reservoir competency would mean fewer bites on hosts with higher reservoir competency (248, 249). Additionally, introducing different types of treatments or altering animal communities can affect tick and tick-borne pathogen composition, as observed in studies of tick abundance and species composition in treated livestock systems in central Kenya (250). Thus, to interrupt disease transmission cycles, IVM programs could include strategies or collaborations to maintain and promote host species richness. However, more research is needed on leveraging the dilution effect for disease risk mitigation, as the numerous and varied complexities within these systems have led to diverse study outcomes (164, 251).

Given these complexities, the dilution effect may not be universally applicable to all geographical locations, vector-borne disease systems, or measures of species richness and infection risk. Such is the case with WNV, the leading cause of arboviral disease in the U.S. (252). Although first recorded domestically in 1999 (253), centuries of human-driven habitat modifications contributed to a decline in overall avian biodiversity while favoring commensal species, many of which are also reservoir hosts for WNV (163). However, the evidence of the relationship between avian species richness and WNV transmission is mixed. For example, in one study avian species richness did not affect WNV prevalence in *Culex* mosquitoes or birds (254), while in another study avian richness was negatively correlated with WNV-positive mosquitoes and human cases but only when assessing non-passerine bird species (255). Promoting and ensuring native species diversity is valuable for a variety of reasons, but the role species diversity plays in IVM programs for disease management is still uncertain. Regardless, effective IVM strategies must account for disease-specific, ecological, and regional factors that can influence disease cycles and impact successful implementation of IVM programs.

### 4.2 Research and collaboration are needed to improve outcomes from educational campaigns

Because many vector-borne diseases do not have an effective approved vaccine to prevent disease manifestation, education on vector bite prevention is vital for preventing vector-borne pathogen transmission. However, increased knowledge on vector-borne diseases may not always correspond with increased personal protection (256–260). This highlights a critical knowledge gap in understanding reasons for initial and long-lasting behavioral change (261).

Educational campaigns rarely assess reductions in bite or disease burden associated with information uptake and behavior change. In the limited number of studies that have evaluated risk reduction through follow-up assessments, the results were surprising. These studies found significant differences in behavior and attitudes toward tick and tick-borne disease prevention. Groups that received educational materials were more likely to adopt preventative measures (91) and reported more positive attitudes, higher levels of knowledge, and greater adoption of tick bite and tick-borne disease prevention methods (92). However, even with these behavior and knowledge changes, both studies reported that there was no significant difference in reported tick bites or tick-borne disease exposure assessed via serology (91, 92). These results suggest that other external social and behavioral factors influence an individual’s assessment of their own risk for exposure to ticks and tick-borne diseases. Collaborations with social and behavioral scientists will be critical in developing and assessing initiatives for consistent and long-lasting risk reduction educational programs.

While vector control educational campaigns may report community knowledge on vectors and vector-borne disease risk, it is vital for these campaigns to follow-up and assess whether the interventions led to a correlated change in entomological indices, reports of vector encounters, or disease incidence. These assessments can be useful in gauging the baseline level of knowledge or beliefs towards preventative practices to optimize future messaging and communication efforts, but further research is required to assess the efficacy of these tools in changing vector and vector-borne disease risk. Additionally, untangling knowledge retention from sustained changes in behavior is complex and will require further studies and long-term monitoring to identify underlying variables that contribute to behavior change.

### 4.3 Costs associated with IVM programs can be prohibitive

Using multiple tools in an IVM plan has been shown to be effective in controlling arthropod vectors, depending on the combination of methods employed. However, there are drawbacks in using multiple control methods, such as high financial costs required to implement and sustain efforts and complexities in determining the true effect of each method to optimize future IVM plans (262). Upfront costs for multiple IVM strategies can be expensive, particularly for large-scale landscape modifications, environmental improvements, and releases of biological control agents. While large-scale modifications and releases are not essential for an IVM program, their implementation has the potential to offset future vector control costs (263).

In general, the more management tactics involved in an IVM plan, the higher the monetary cost. Unfortunately, the projected costs to run vector control programs and to implement multiple control efforts are not always aligned with current budgets or people’s willingness to pay for vector control (264). However, studies in the U.S. found that communities that experience higher mosquito population densities or disease outbreaks may be more willing to pay for mosquito abatement programs. For instance, a study in New Jersey found that respondents were willing to pay on-average three times the current operating costs for mosquito abatement in their district (265). Studies in Arizona, Florida, and Texas also showed increased willingness to pay for mosquito control efforts, further emphasizing the desire for expanded mosquito control (266, 267). This willingness to pay for vector control is not true for all vector systems. An integrated tick control program would cost between $508-3,192 annually per household; however, a survey found that people were only willing to pay $100–150 annually for tick control (262). Furthermore, costs for control efforts can rise exponentially depending on the size of the land and application frequency required to control ticks.

The cost of paying for expanded services offered by vector control programs is likely to fall on citizens through tax increases (264). However, when asked if the public would support tax increases to pay for extra tick control services, only 21% of responding U.S. vector control programs believed that the public would support the tax increase, with the highest support in the Northeast and the lowest support in the Upper Midwest (264). Unsurprisingly, as the risk of disease increases, so does the willingness to pay for vector control programs. In Wisconsin, residents were initially only willing to pay for nuisance mosquito control, but when WNV disease risk increased so did their willingness to pay for mosquito control services targeting these vectors (268).

When compared to other management tactics, a biological control component can be an expensive tool due to high resource and economic costs. High host specificity of the biological control agent can limit the production speed of these agents to adequately meet demand and need. Biological control agents also require resources for growth and maintenance in optimal conditions. Furthermore, some biological control agents require inundative releases to maintain population levels at a density that can adequately reduce the arthropod vector abundance (114, 124, 133). The use of genetic manipulation, such as through gene editing or gene drive insertion to reduce vector populations or through selective breeding programs and genetic modification for adaptive or resistant traits in livestock, can be effective but is extremely expensive in initial stages of research and development, and often remains expensive in application but holds promise with future advances and cost reductions (128, 130). Ultimately, many biological control options can be costly and economically limiting when considering the knowledge, time, and resources required to rear, release, and maintain biological control populations and when using genetic modification tools and strategies (114, 120, 123, 124, 130).

Lastly, while cooperative planning and specialized knowledge on local vectors and vector-borne diseases are paramount for developing and deploying effective IVM plans, this is not always feasible due to resource limitations. For instance, many locations with high vector-borne disease burden may face challenges due to limited personnel or expertise, time and resource constraints, or limited buy-in from municipal, organizational, or community partners (263, 269). As communities develop their ideal IVM plans, they should be realistic with their goals based on the additional cost, resources, and time required to implement several methods.

## 5 Conclusion

Vector-borne diseases have far-reaching effects, including direct loss of life or livelihood, economic impacts from mortality or reduced agricultural production, and both direct and indirect consequences for conservation, wildlife management, and ecosystem services. Vector-borne disease systems are highly complex and not yet fully understood. However, strategies for managing vectors and their associated diseases are continually evolving, prioritizing flexible and adaptable approaches rather than absolute control. Integrated vector management principles offer a future-oriented framework for addressing vector-borne diseases across the three interconnected spheres of the One Health paradigm—human, animal, and environmental health.

As new strategies are developed, they should be rigorously evaluated and tailored to specific ecological contexts, ensuring effective and sustainable outcomes. Data-driven decision-making, supported by ongoing research and case studies, will remain critical for refining practices and guiding more successful iterations of management in the future. Furthermore, future work should focus on the economic and efficacious impacts when implementing integrative strategies and comparisons across different plans and geographical regions, thereby providing communities with information to make informed decisions about applying IVM strategies within a One Health context. The principles underlying both One Health and IVM have evolved over time into robust paradigms that align closely in their goals of promoting the long-term health of animals, people, and the environment. Though not always discussed in tandem, these frameworks share a commitment to addressing the complex challenges posed by vector-borne diseases in a holistic and integrative manner.
